# Optimal Nutritional Status for a Well-Functioning Immune System Is an Important Factor to Protect against Viral Infections

**DOI:** 10.3390/nu12041181

**Published:** 2020-04-23

**Authors:** Philip C. Calder, Anitra C. Carr, Adrian F. Gombart, Manfred Eggersdorfer

**Affiliations:** 1 Faculty of Medicine, University of Southampton and NIHR Southampton Biomedical Research Centre, University Hospital Southampton NHS Foundation Trust, Tremona Road, Southampton SO16-6YD, UK; P.C.Calder@soton.ac.uk; 2 Nutrition in Medicine Research Group, Department of Pathology & Biomedical Science, University of Otago, Christchurch, P.O. Box 4345, Christchurch 8140, New Zealand; anitra.carr@otago.ac.nz; 3 Linus Pauling Institute, Department of Biochemistry and Biophysics, Oregon State University, 307 Linus Pauling Science Center, Corvallis, OR 97331, USA; adrian.gombart@oregonstate.edu; 4 Department of Internal Medicine, University Medical Center Groningen, 9713 GZ Groningen, The Netherlands

**Keywords:** immune system, viral infection, influenza, COVID-19, micronutrients, vitamins, omega-3 fatty acids, minerals, vitamin C, vitamin D

## Abstract

Public health practices including handwashing and vaccinations help reduce the spread and impact of infections. Nevertheless, the global burden of infection is high, and additional measures are necessary. Acute respiratory tract infections, for example, were responsible for approximately 2.38 million deaths worldwide in 2016. The role nutrition plays in supporting the immune system is well-established. A wealth of mechanistic and clinical data show that vitamins, including vitamins A, B_6_, B_12_, C, D, E, and folate; trace elements, including zinc, iron, selenium, magnesium, and copper; and the omega-3 fatty acids eicosapentaenoic acid and docosahexaenoic acid play important and complementary roles in supporting the immune system. Inadequate intake and status of these nutrients are widespread, leading to a decrease in resistance to infections and as a consequence an increase in disease burden. Against this background the following conclusions are made: (1) supplementation with the above micronutrients and omega-3 fatty acids is a safe, effective, and low-cost strategy to help support optimal immune function; (2) supplementation above the Recommended Dietary Allowance (RDA), but within recommended upper safety limits, for specific nutrients such as vitamins C and D is warranted; and (3) public health officials are encouraged to include nutritional strategies in their recommendations to improve public health.

## 1. Introduction

Acute respiratory tract infections are a major cause of morbidity and mortality across the globe, as illustrated by both seasonal influenza epidemics, and the recent outbreak of the coronavirus disease, COVID-19, caused by SARS-CoV-2 infection. The World Health Organization (WHO) estimates that worldwide, seasonal influenza alone results in 3–5 million cases of severe illness that require hospitalization, and 290,000–650,000 deaths annually [1]. In aggregate, acute respiratory tract illnesses were estimated to be responsible for approximately 2.38 million deaths worldwide in 2016 [2,3]. Indeed, severe lower respiratory tract infections were the most common cause of sepsis-related death globally from 1990–2017 [4].

A number of standard public health practices have been developed to help limit the spread and impact of respiratory viruses, such as regular hand washing, avoiding those showing symptoms of infection, and covering coughs [5]. For certain viruses, such as influenza, annual vaccination campaigns designed to prime the immune response in case of exposure exist in many countries. Influenza is caused by a single-stranded RNA virus, and as such exhibits high mutation rates and rapid evolution, which may allow these viruses to escape from pre-existing neutralizing antibodies in the host [6]. Vaccination programs therefore must make predictions each year as to which strains to vaccinate against, with varying degrees of success. In the US, the Centers for Disease Control and Prevention estimate the current year influenza vaccine to be 45% effective for preventing medically attended, laboratory-confirmed influenza virus. This is consistent with estimates from the previous years when the influenza vaccines were antigenically matched to the circulating viruses [7]. Since the 2011–2012 season, vaccine efficacy has ranged from 19%–54% [8].

The immune system is comprised of both the innate (fast, non-antigen specific) and adaptive (slower, antigen-specific) responses. The innate immune system is comprised of physical barriers that help prevent pathogen entry (e.g., skin, gut epithelium), antimicrobial peptides, the complement system, and a variety of phagocytic and other cells (e.g., neutrophils, macrophages, natural killer cells), that recognize the presence of pathogens via the expression of nonspecific pattern-recognition receptors [9]. The innate system moves quickly to recognize and destroy “non-self” threats, typically via inflammatory processes, and then resolve the inflammation and repair the damage caused by these events [9]. However, innate immunity does not increase efficacy or speed of response with repeated exposure to a pathogen. Subsequent to the innate response, the adaptive response is engaged. The adaptive response includes antigen-specific cells, e.g., T lymphocytes, subsets of which coordinate the overall adaptive response or kill virally-infected cells, and B lymphocytes, which can be activated to secrete antibodies specific to the infecting pathogen [9]. While slower to respond than the innate system, the adaptive system is responsible for generating immunological “memory”, whereby a repeated infection with the same pathogen will generate a vigorous, fast antigen-specific response [9]. The induction of immunological memory is the mechanism by which vaccines can provide protection against subsequent pathogen exposure.

Undoubtedly, public hygiene practices and, when available, vaccinations can be effective mechanisms to provide protection against infectious disease. However, vaccines can take years to create, are not available against all viruses (including the current coronavirus SARS-CoV-2), and provide varying levels of protection. The morbidity and mortality numbers cited above highlight the need for additional strategies to support the immune system, in order to reduce the impact of respiratory and other infections.

## 2. Nutritional Impact on Immunity

Often missing in public health discussions around immunity and infection are nutritional strategies to support optimal function of the immune system. This is surprising, given that the importance that nutrition plays in immune function is well established. Several vitamins, including vitamins A, B_6_, B_12_, C, D, E, and folate; and trace elements, including zinc, iron, selenium, magnesium, and copper, play important and complementary roles in supporting both the innate and adaptive immune systems. Deficiencies or suboptimal status in micronutrients negatively affect immune function and can decrease resistance to infections [10,11,12]. Indeed, with the exceptions of vitamin E and magnesium, each of these micronutrients has been granted health claims in the European Union for contributing to the normal function of the immune system [13]. Other nutrients such as omega-3 fatty acids also support an effective immune system, specifically by helping to resolve the inflammatory response [14].

The mechanistic roles that micronutrients play to optimize immune function have been well-described recently [10,12]. Most micronutrients exhibit pleiotropic roles in supporting immune function. With respect to innate immunity, the vitamins and minerals listed above collectively function to support the development and maintenance of physical barriers; production and activity of antimicrobial proteins; growth, differentiation and motility/chemotaxis of innate cells; phagocytic and killing (e.g., oxidative burst) activities of neutrophils and macrophages; and promotion of and recovery from inflammation (e.g., cytokine production and antioxidant activity). They also support adaptive immunity, via lymphocyte differentiation, proliferation and homing; cytokine production; antibody production; and the generation of memory cells. The roles that vitamins C and D play in immunity are particularly well elucidated. Vitamin C affects several aspects of immunity, including supporting epithelial barrier function, growth and function of both innate and adaptive immune cells, white blood cell migration to sites of infection, phagocytosis and microbial killing, and antibody production [10]. Many immune cells have vitamin D receptors that affect their function after ligand binding, and as such vitamin D profoundly influences immunity. For example, it promotes differentiation of monocytes to macrophages and increases their killing capacity; modulates the production of inflammatory cytokines; and supports antigen presentation. Furthermore, vitamin D metabolites appear to regulate production of specific antimicrobial proteins that directly kill pathogens, and thus are likely to help reduce infection including in the lungs [15,16].

As mentioned above, inflammation is a key component of the immune response. This response is caused by a variety of pro-inflammatory mediators, produced by several different types of cells, resulting in the influx of fluid, immune cells, and other mediators that function to eliminate the infection. Inflammation typically resolves quickly at the end of the immune response, due to activation of specific negative-feedback mechanisms. Among these, the omega-3 fatty acids, eicosapentaenoic acid (EPA) and docosahexaenoic acid (DHA) present at the site of inflammation are enzymatically converted to specialized pro-resolving mediators (SPMs) known as resolvins, protectins, and maresins. These molecules, along with others, function together to orchestrate the resolution of inflammation and to support healing, including in the respiratory tract [14,17]. Notably, nutritional deficiencies in these essential fatty acids can result in delayed or suboptimal resolution of inflammation [17]. This could be very important in the context of severe COVID-19 which manifests as uncontrolled inflammation, the so-called cytokine storm [18,19], linked with acute respiratory distress syndrome (ARDS). A number of the SPMs formed from EPA and DHA have been shown in animal models to both protect against and resolve acute lung injury and ARDS [20,21,22,23,24]. Nutritional formulas containing antioxidants and rich in EPA and DHA have been used in several human trials of patients with ARDS. A recent Cochrane review of these trials identified a significant improvement in blood oxygenation and significant reductions in ventilation requirement, new organ failures, length of stay in the intensive care unit and mortality at 28 days [25]. Taken together, these findings suggest an important role for EPA and DHA in ameliorating inflammation and lung injury, perhaps acting via conversion to SPMs.

It is not surprising, then, that deficiencies and even suboptimal status of these nutrients can impair immune functions. Depending on the deficient nutrient or nutrients, there can be decreases in the numbers of lymphocytes, impairment of phagocytosis and microbial killing by innate immune cells, altered production of cytokines, reduced antibody responses, and even impairments in wound healing [12]. These functional impairments are, presumably, what lead to the clinical immune-related manifestations of deficiency. Indeed, people deficient in vitamin C are susceptible to severe respiratory infections such as pneumonia [10,26]. A recent meta-analysis reported a significant reduction in the risk of pneumonia with vitamin C supplementation, particularly in individuals with low dietary intakes [27]. In older patients, disease severity and risk of death were reduced with supplementation, particularly in the case where initial plasma levels of vitamin C were low [27]. Vitamin C supplementation has also been shown to decrease the duration and severity of upper respiratory tract infections, such as the common cold, and significantly decrease the risk of infection when given prophylactically in people under enhanced physical stress [26,28].

Likewise, vitamin D deficiency increases the risk for respiratory infection. Observational studies report an association between low blood concentrations of 25-hydroxyvitamin D (the major vitamin D metabolite) and susceptibility to acute respiratory tract infections [29,30]. Consistent with these findings, several recent meta-analyses have concluded that vitamin D supplementation can reduce the risk of respiratory tract infections in both children and adults [11,31,32,33,34,35]. In 2017, Martineau and colleagues performed a systematic review and meta-analysis of individual participant data (*n* = 10,933) from 25 randomized, double blind, placebo controlled trials of vitamin D supplementation with a specified outcome of acute respiratory tract infection (ARI). They found a 12% reduction for experiencing at least one ARI irrespective of dosing schedule [11]. They found a 19% reduction in individuals taking a daily or weekly dose without bolus doses and no benefit with bolus dosing. Among those receiving a daily or weekly dose, they observed a 25% reduction for those with baseline 25(OH)D levels ≥25 nmol/L (12 ng/mL) and a 70% reduction for those with baseline levels <25 nmol/L [23]. They concluded that daily or weekly vitamin D supplementation protected against ARI overall and that it was safe.

Clinical outcomes also demonstrate a role for vitamin E in respiratory tract infections. In a randomized controlled trial of 617 nursing home residents, daily supplementation for one year with 200 IU vitamin E reduced the risk of upper respiratory tract infections, but not lower respiratory tract infections [36]. Vitamin E enhances T cell-mediated immune function in the face of age-related decline [37]. In one study, supplementation of older adults with vitamin E improved natural killer cell activity, neutrophil chemotaxis and phagocytosis, and mitogen-induced lymphocyte proliferation [38]. In a second study, vitamin E supplementation improved T cell-mediated immunity as measured by increased production of antibodies to hepatitis B virus and tetanus vaccines [39].

Finally, marginal zinc deficiency can also impact immunity. Zinc is important for maintenance and development of cells in both the innate and adaptive immune systems. Zinc deficiency results in impaired formation, activation and maturation of lymphocytes, disturbs the intercellular communication via cytokines, and weakens the innate host defense [40,41]. Those deficient in zinc, particularly children, are prone to increased diarrheal and respiratory morbidity [42,43].

Furthermore, data from animal models and epidemiological studies in people indicate that deficiency in specific nutrients, particularly selenium and vitamin E, can lead to reproducible genetic mutations and increased virulence of certain viruses, including coxsackievirus, poliovirus, and murine influenza [44,45]. In a double-blind placebo controlled study, an increase of selenium intake by otherwise healthy subjects with relatively low levels of plasma selenium concentrations improved cellular immunity. Subjects receiving selenium cleared an oral live attenuated poliomyelitis vaccine more rapidly and sequence analysis of the viral genome showed lower numbers of mutations as compared to those receiving the placebo. These data suggest that suboptimal nutrient status in the host population could lead to the emergence of more pathogenic strains of viral diseases, thereby increasing the risks and burdens associated with these illnesses. Given the current situation, it may be beneficial to further pursue this line of investigation.

Optimal intake of all these nutrients ideally would be achieved through the consumption of a well-balanced and diverse diet, but this can be difficult to accomplish for the general population. Indeed, it is generally accepted that nutrient inadequacies and deficiencies are widespread [46,47,48,49,50] (and references therein). Biochemical markers of nutrient status are particularly useful in assessing inadequacy or deficiency, and lead to the conclusion that intakes often are not sufficient. For example, extensive data have been published, using blood 25-hydroxyvitamin D levels to assess vitamin D status. A systematic review involving 195 studies in forty-four countries reported that 37.3% of the studies found mean values lower than 50 nmol/L [51]. The US Institute of Medicine (IOM) committee that reviewed Dietary Reference Intakes (DRI) for vitamin D has suggested that those with concentrations less than this level are at risk for inadequacy, while those with concentrations between 50–75 nmol/L are considered sufficient [52,53]. Interestingly, while the highest vitamin D levels were reported in North America, data from the United States still indicate that 8% of the non-infant population was at risk for vitamin D deficiency, and 17% exhibited concentrations below the 25(OH)D level that is associated with desirable intake [53]. Other studies based on 25(OH)D levels indicate that vitamin D inadequacy or deficiency are also prevalent in Europe and China [54,55,56]. Similarly, a recent systematic review involving 132 studies of serum alpha-tocopherol status indicated that 13% of the values were below the threshold of deficiency (12 mmol/L). Deficiency was noted in the Americas, Asia Pacific, Europe, the Middle East and Africa [57]. The situation with vitamin C is similar. Currently, the most commonly used vitamin C cutoff levels are approximately ≤23–28 µmol/L for hypovitaminosis C and ≤11 µmol/L for deficiency [58]. The evidence indicates that vitamin C insufficiency or deficiency is common in low and middle-income countries (e.g., Mexico, Brazil, India), and not uncommon in high income countries (e.g., US, Singapore, New Zealand), particularly in at-risk subpopulations [53,59,60,61,62,63,64,65,66,67]. Furthermore, the WHO and the Food and Agriculture Organization (FAO) of the United Nations have described that, based on blood markers, vitamin A and iron deficiencies are widespread and of significant global concern [46,49,50]. Status data in the general population or specific subpopulations also reveal inadequacies or deficiencies in various countries, including in developed nations, for vitamins B6, B12, and folate, as well as zinc and selenium [53,59,60,68,69,70,71,72,73]. Finally, a global survey of EPA + DHA status in the blood, from 298 studies, found “low” or “very low” status (i.e., levels associated with increased risk of cardiovascular related mortality) of EPA + DHA in most of the countries assessed [74]. Collectively, the totality of these data strongly suggest that micronutrient and omega-3 inadequacies or deficiencies are prevalent around the globe.

It should also be noted that optimal nutritional support for the immune system can require intakes above the RDA for some micronutrients, while at the same time infections and other stressors can reduce micronutrient status in the body. Vitamin C levels, in particular, decrease during times of infection and higher intakes are required to restore normal blood levels [10,75]. These higher intakes and blood levels are associated with improved clinical outcomes. For example, supplementation of pneumonia patients with ≥200 mg/d vitamin C restored depleted plasma and cellular vitamin C levels, and resulted in decreased respiratory symptom scores and a dose-dependent decrease in hospital length of stay [76,77].

## 3. Recommendations and Conclusions

Thus, a set of clear nutritional recommendations is needed (Table 1). First, supplementation with micronutrients and omega-3 fatty acids is a safe, effective, and low-cost way to help eliminate nutritional gaps and support optimal immune function, and therefore reduce the risk and consequences of infections [10,12]. Intakes should follow recommended upper safety limits set by expert authorities, such as the European Food Safety Authority and, in the United States, the IOM. Thus, a multivitamin and mineral supplement that supplies the basic micronutrient requirements (e.g., RDA) for vitamins and minerals is recommended in addition to the consumption of a well-balanced diet.

Second, we recommend supplementation above the RDA for vitamins C and D. As noted above, recent meta-analyses concluded significant reductions in the risk and impact of both upper and lower respiratory tract infections such as the common cold and pneumonia, including disease severity and risk of death in older patients, with vitamin C supplementation [27,28,79]. Based on this evidence, a daily intake of at least 200 mg/day for healthy individuals is recommended. This level is above the US RDA of 75 and 90 mg/day for female and male adults, respectively [80]. It should be noted that vitamin C requirements depend on health status, and 1–2 g/day are recommended to restore normal blood levels in individuals who are sick, beginning at the onset of symptoms. These levels are within the US tolerable upper limit (TUL) for adults of 2 g/day (note that the upper limit for children aged 1–3 years is 400 mg/day) [80].

Several recent meta-analyses have concluded that vitamin D supplementation reduces the risk of respiratory tract infections in both children and adults [11,31,32,33,34,35]. Protective effects were seen with those receiving daily or weekly vitamin D, but not with less frequent bolus doses [11,32]. A daily intake of 2000 IU (50 µg) is recommended. This is above the US RDA of 400–800 IU (depending on age), but below the TUL for those over 1 year of age (2500–4000 IU) [52].

A third recommendation involves the omega-3 fatty acids EPA and DHA. An adequate intake supports the resolution of inflammation via the production of anti-inflammatory metabolites of these fatty acids, including in the respiratory tract [14,17]. An intake of 250 mg EPA + DHA per day is recommended, consistent with global, regional and national expert recommendations [81,82,83].

Public health practices, such as vaccinations and hygiene measures, are important measures that help limit the spread and impact of infections, including against acute respiratory viruses. However, the present situation with SARS-CoV-2 infection and severe outcomes of COVID-19 and the annual morbidity and mortality figures for respiratory infections overall make it clear that these practices alone are not sufficient. New strains of influenza continuously emerge, necessitating development of new vaccines with varying efficacy, and outbreaks of novel viruses can be enormously difficult to contain. As such, additional safe and cost-effective strategies are needed to support the immune system, and further protect individuals and populations from harm. One compelling strategy is to provide sufficient nutritional support for the immune system. As described above, optimal nutrient intake, including supplementing above the RDA for certain immune-supporting vitamins, promotes optimal immune function, helps to control the impact of infections, and could help limit the emergence of novel, more virulent strains of pathogenic viruses. We, therefore, strongly encourage public health officials to also include nutritional strategies in their arsenal to improve public health and to limit the impact of seasonal and emerging viral infections.

## Figures and Tables

**Table 1:** Recommended intakes of selected nutrients to support optimal immune function.

Nutrient	Rationale	Recommendation
Vitamins and trace elements	These micronutrients play important roles in supporting the cells and tissues of the immune system. Deficiencies or suboptimal status in these micronutrients negatively affect immune function and can decrease resistance to infections.	A multivitamin and trace element supplement that supplies the nutrient requirements (e.g., 100% US RDA for age and gender) [78] for vitamins and trace elements including vitamins A, B_6_, B_12_, C, D, E, and folate, and trace elements including zinc, iron, selenium, magnesium and copper. This is in addition to the consumption of a well-balanced diet.
Vitamin C	Doses of ≥200 mg/day provide saturating levels in the blood, and support reduction in the risk, severity and duration of upper and lower respiratory tract infections. Requirements for vitamin C increase during infection.	Daily intake of at least 200 mg/day for healthy individuals. In individuals who are sick, 1–2 g/day is recommended.
Vitamin D	Daily supplementation of vitamin D reduces the risk of acute respiratory tract infections.	Daily intake of 2000 IU/day (50 µg/day).
Zinc	Marginal zinc deficiency can impact immunity. Those deficient in zinc, particularly children, are prone to increased diarrheal and respiratory morbidity.	Daily intake in the range of 8–11 mg/day.
Omega-3 fatty acids (EPA + DHA)	Omega-3 fatty acids support an effective immune system, including by helping to resolve inflammation.	Daily intake of 250 mg/day of EPA + DHA.
